# Medical Education for “Generation Z”: Everything online?! – An analysis of Internet-based media use by teachers in medicine

**DOI:** 10.3205/zma001168

**Published:** 2018-05-15

**Authors:** Markus Vogelsang, Katrin Rockenbauch, Hermann Wrigge, Wolfgang Heinke, Gunther Hempel

**Affiliations:** 1 University of Leipzig, Department of Anesthesiology and Intensive Care Medicine, Leipzig, Germany; 2 University of Leipzig, Vice-Rectorate for Education and International Affairs, Project: Teaching practice in transfer, Leipzig, Germany; 3 District of Mittweida Hospital gGmbH, Department for Anesthesiology and Interdisciplinary Intensive Care Medicine, Mittweida, Germany

**Keywords:** Social media, Internet, Medical education, Internet-based teaching, Germany

## Abstract

**Aim: **The aims of this study were to gain an overview of the web-based media used during the clinical phase of medical study at German medical schools and to identify the resources needed for web-based media use. Also examined were the influences on web-based media use, for instance, the assessment of their suitability for use in teaching.

**Method: **An online survey of 264 teacher coordinators in internal medicine, surgery, anesthesiology, gynecology, pediatrics and psychiatry was conducted in March and April, 2016. This survey was carried out in the German-speaking countries using a 181-item questionnaire developed by us. Analysis took place in the form of descriptive and exploratory data analysis.

**Results: **The response rate was 34.8% with 92 responses. Individual web-based media were actively used in the classroom by a maximum of 28% of participants. Reasons cited against using web-based media in teaching included the amount of time required and lack of support staff. The assessment of suitability revealed that interactive patient cases, podcasts and subject-specific apps for teaching medicine were predominantly viewed as constructive teaching tools. Social media such as Facebook and Twitter were considered unsuitable. When using web-based media and assessing their suitability for teaching, no correlations with the personal profiles of the teachers were found in the exploratory analysis, except regarding the use of different sources of information.

**Conclusion:** Despite the Internet’s rapid development in the past 15 years, web-based media continue to play only a minor role in teaching medicine. Above all, teacher motivation and sufficient staff resources are necessary for more effective use of Internet-based media in the future.

## Zusammenfassung

**Zielsetzung: **Ziel dieser Untersuchung war es einen Überblick über den Einsatz von Onlinemedien im klinischen Studienabschnitt der humanmedizinischen Lehre deutschsprachiger medizinischer Fakultäten zu bekommen, dafür notwendige Ressourcen zu identifizieren, sowie Einflüsse auf die Nutzung von Onlinemedien, wie die Einschätzung der Lehreignung, zu erfassen.

**Methodik: **Von März bis April 2016 wurde hierfür eine Onlinebefragung unter 264 Lehrverantwortlichen der Fachrichtungen Innere Medizin, Chirurgie, Anästhesiologie, Gynäkologie, Pädiatrie und Psychiatrie im deutschsprachigen Raum durchgeführt. Die Befragung erfolgte mit Hilfe eines selbst erstellten Fragebogens, der 181 Items erfasste. Die Auswertung erfolgte über eine deskriptive und explorative Statistik.

**Ergebnisse: **Die Rücklaufquote lag mit 92 Rückmeldungen bei 34,8%. Die einzelnen Onlinemedien wurden von maximal 28% der Teilnehmenden aktiv im Unterricht eingesetzt. Als Gründe gegen eine Verwendung von Onlinemedien im Unterricht wurden die benötigte Arbeitszeit und mangelnde personelle Unterstützung genannt. Die Bewertung der Lehreignung ergab, dass vor allem interaktive Onlinefallbearbeitungen, Podcasts und fachspezifische Apps für die Lehre in der Humanmedizin als geeignet angesehen wurden. Soziale Medien wie Facebook und Twitter wurden als ungeeignet eingeschätzt. Bei der Verwendung von Onlinemedien und der Bewertung der Lehreignung zeigten sich in der explorativen Statistik, bis auf die Nutzung verschiedener Informationsquellen, keine Zusammenhänge zu persönlichen Merkmalen der Lehrenden.

**Schlussfolgerung: **Trotz der rasanten Entwicklung des Internets in den letzten 15 Jahren spielen Onlinemedien für das Lehrgeschehen in der Humanmedizin weiterhin nur eine untergeordnete Rolle. Um Onlinemedien künftig effektiver zu nutzen werden vor allem eine hohe Motivation der Lehrenden und ausreichende personelle Ressourcen benötigt.

## 1. Introduction

The present generation of medical students was mostly born after 1995 and is familiar with digital media as a matter of course. People of this age are often referred to as “Generation Z” even though there is growing criticism of this label [1], [2], [3]. According to a current meta-analysis, approximately 70-80% of surveyed students use social media on a daily basis and about 20% of these use them for study purposes [4]. Each student spends an average of 30-40 minutes a day with Facebook alone [5]. This platform is used independently by medical students to prepare for exams, share online-based materials, organize meetings and exchange important information [6]. Even among teachers the use of these modern media is no longer unusual [7], [8]. Teachers use social media to establish and maintain contact with colleagues, share knowledge and engage in continuing education [8]. Overall, new and easy-to-use options for interacting with like-minded peers are playing a major role [9], [10]. This trend is intensified by the presence of many mobile devices, such as smartphones, tablets and laptops, which enable access to the Internet from anywhere and at any time [11]. In higher education this digital revolution also represents new opportunities for using Internet-based media [12], [13]. The online environment already exerts great influence on education [6], [14], [15], [16], [17]. In their review, Hollinderbäumer et al. see the inclusion of Web 2.0 and social media as the current form of self-directed learning [16]. Since 2010 there has been an increase in publications giving examples for using various social media in medical education [16]. Moreover, it has also been shown that a university’s course offerings significantly influence the diversity of web-based media use among students [3]. However, the pertinent literature on user statistics and opportunities comes primarily from the Anglo-American countries [16]. To develop common strategies among German-speaking medical schools for web-based media use in medical education, the current situations at individual medical schools should be explored. To accomplish this, the following questions should be answered concretely:

1. How widely established was the use of Internet-based media during the clinical phase of medical study in 2016?
2. Which resources for using Internet-based media should be available to teachers?
3. Are there personal characteristics among teachers that positively or negatively affect the use of Internet-based media?

### Definition of terms

The definition of “Internet-based media“ in this paper is based on the definitions published in O’Reilly (2007) and Sangra et al. (2012) [18], [19]. We include both commonly known forms of e-learning (interactive patient cases, web-based teaching materials, etc.) and innovations associated with Web 2.0 (Facebook, instant messaging, cloud computing, podcasts and online encyclopedias).

## 2. Methods

To answer the research questions, an online survey was conducted in March and April, 2016, on the platform Sosci Survey (https://www.soscisurvey.de/). The participant address list was generated through an Internet search of the university hospital websites by the authors. A total of 264 teacher coordinators at 44 university hospitals in Germany, Austria and Switzerland were invited to take the survey. The selection of the medical schools was based on the membership of the Medizinischer Fakultätentag with the inclusion of Basel, Bern, Graz, Innsbruck, Salzburg, Vienna and Zurich [20]. This was done to facilitate a comparison of the results by country. Since course offerings and the focus placed on individual medical subjects can vary widely among medical schools, the size of a professional category—as an independent criterion—was used to select subjects. Data collection was done under the six largest professional categories (internal medicine, surgery, anesthesiology, gynecology, pediatrics and psychiatry) with a primary focus on inpatient care [21]. To allow for better comparison of the responses, no pre-clinical subjects were included due to structural differences between them and the clinical subjects. A web-based tool enabling address correction was used to ensure that all participants received the invitation to participate. As a result, all 264 teachers were sent up to three emails inviting them to take part in the survey.

To consider more precisely the issue of web-based media use and the influences on teachers’ use, the following hypotheses were formulated and tested based on the survey responses:

- The use of social media differs significantly from the use of other Internet-based media.
- A significant difference exists between using Internet-based media in the classroom and using Internet-based media to make materials available for self-study.
- There is a correlation between the information sources used regarding Internet-based media and the use of these media in the classroom.
- A correlation exists between personal use of Internet-based media and the assessment of suitability for teaching purposes.

The survey was conducted using a questionnaire designed by us focusing on these hypotheses (see attachment 1 ). The questionnaire breaks down into:

- Five questions about personal and professional information;
- 28 items addressing personal use and basic attitudes toward web-based media based on a questionnaire by Pscheida et al. [7];
- Nine questions about use of web-based media for educational purposes:
- Seven questions on organizational and staff resources in each subject area;
- Six questions on teacher coordination and teaching materials in each subject area.

To record personal use and basic attitudes toward web-base media, the validated questionnaire from a study by Pscheida et al. was used and adapted as needed [7]. The basic attitude toward web-based media was divided into four dimensions: “openness,” “fear,” “concern about privacy,” and “confidence” [22]. Five- and seven-point Likert scales and checkbox systems with a multiple answer function were used in the survey. A sliding scale (from one to 100) was used to assess the suitability of individual web-based media for teaching purposes, the evaluation of “obstacles,” and to indicate the work involved. There was an option to write open-ended comments. To verify the questionnaire’s functionality and understandability, we carried out a pre-test among university teachers of various subjects at different university hospitals in October and November, 2015, and included the knowledge gained from that in the final version of the questionnaire. Once the survey was over, the responses were checked for completeness using Sosci Survey’s function for weighting missing answers and counting the number of pages answered by the participants. The program SPSS provided by IBM was used for the subsequent data analysis. The Shapiro-Wilk test was used to verify normal distribution as a test prerequisite [23]. Spearman’s rank-order correlation (correlation coefficient *r_s_* [number of responses included]) was used to check for correlations on the ordinal scale. The level of significance was defined as p<0.05.

### Data privacy

The survey was reviewed and approved by the University of Leipzig’s data protection officer. The questionnaires were not broken down by individual medical schools in order to protect the anonymity of the participants.

## 3. Results

### Sample description

The response rate was 34.8% (N=92). After eliminating incomplete data sets, N=83 questionnaires were included in the analysis. The analysis showed differences in the percentage of subject areas: while only 1.2% of the 83 responses came from internal medicine, the percentages of the other subject areas ranged from 14.5% (surgery) to 27.7% (anesthesiology). Due to the small sample of participants from outside Germany, there was no comparison of countries in the analysis. The majority of those surveyed (N=60; 65.2%) reported belonging to a medical school following the conventional curriculum; 21 (21.8%) reported belonging to an institution with a model curriculum; and for two participants this information was unknown. A total of 48 (58%) participants indicated their age to be 40-59 years. Thirty-one (37%) were between 20-39 years old and four (5%) between 60-69. Teaching qualifications and the information sources consulted on web-based media are listed in table 1 (Tab. 1). It was shown that the more information sources a teacher consulted (rs(83)=0.484; p<0.01), the more the teacher used Internet-based media in the classroom. Teaching qualifications, in contrast, had no influence on this use. The participants’ personal use of web-based media is shown in table 2 (Tab. 2).

#### Basic attitudes toward Internet-based media

**Figure 1:**
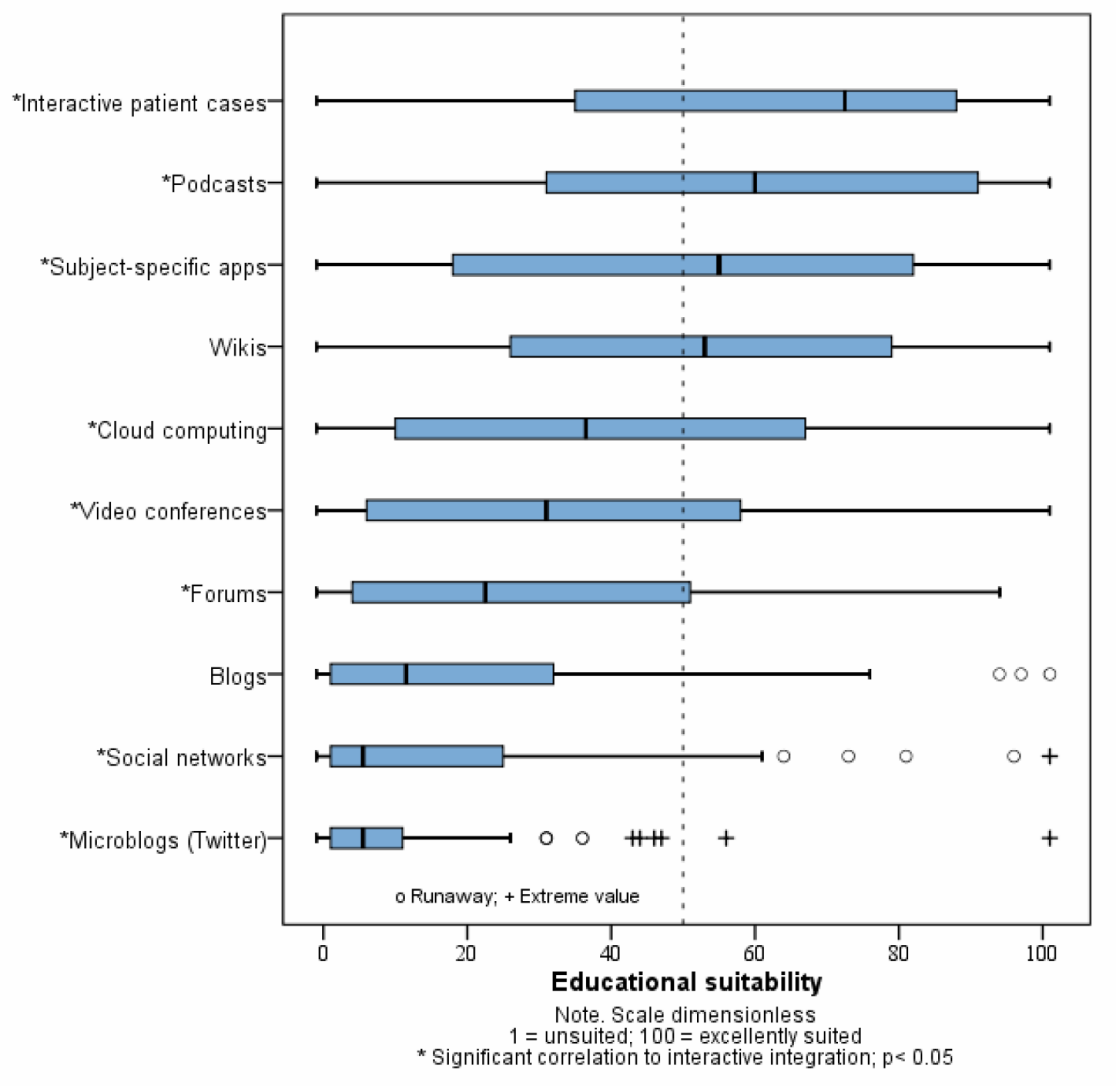
Assessment of the educational suitability of individual Internet-based media

The basic attitude of teachers toward web-based media was measured on a five-point ordinal scale (5=agree completely; 1=disagree completely). Attitudes were described as being more open than otherwise (median=3.5; mean=3.47; SD=1.1) and without fear in regard to potentially incorrect use (median=2; mean=2.17; SD=0.90). Participants also assessed themselves as being confident in their use of web-based media (median=4; mean=3.93; SD=0.81). No clear tendency could be discerned in respect to concerns about privacy (median=3.17; mean=3.07; SD=0.78). The assessment of individual Internet-based media as being suitable for teaching purposes is presented in figure 1 (Fig. 1). Interactive patient cases, podcasts and subject-specific apps were considered to be particularly suitable for teaching and learning, whereas social media such as Facebook, Twitter and blogs were viewed as very unsuitable. The basic advantages and disadvantages of using web-based media according to the teachers and reasons against using them are given in table 3 (Tab. 3). Improved diversity of teaching strategies and a higher level of student satisfaction regarding the course offerings were seen as advantages. In contrast, greater amounts of required time and effort along with a lack of support staff emerged as obstacles.

#### Internet-based media in teachers’ daily lives

**Figure 2:**
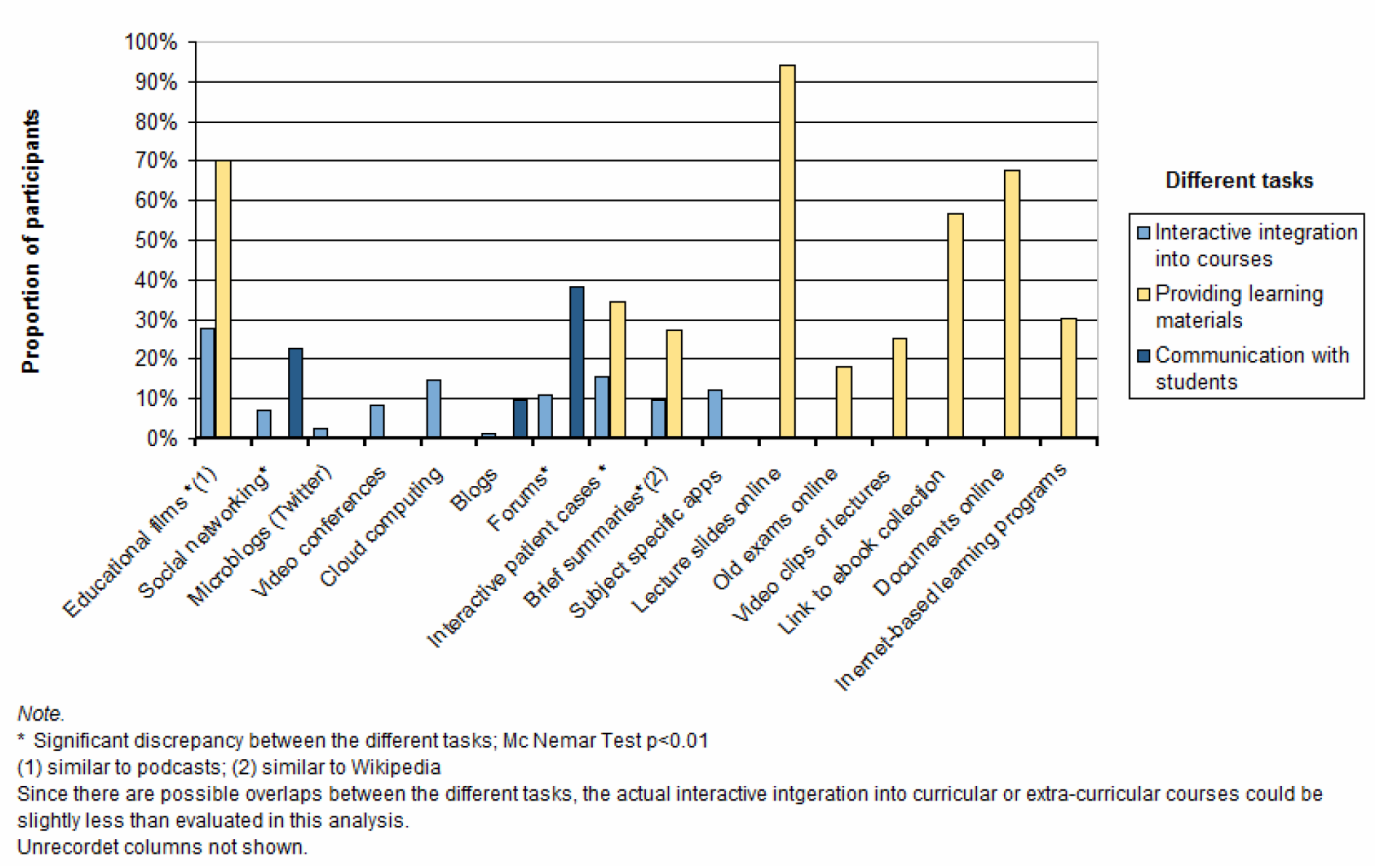
Teachers' use of Internet-based media according to the different tasks

When considering teachers’ use of web-based media, we differentiated between providing learning materials for self-study, communicating electronically with students, and interactive integration into curricular or extra-curricular courses. In figure 2 (Fig. 2), web-based media use is presented according to these three tasks and points of overlap in the use of such media are compared using McNemar’s test for paired data. About half of the teachers (50.6%; N= 42) actively use at least one Internet-based medium in the classroom. Eight participants reported teaching courses in which the use of web-based media is mandatory. This mainly involved interactive patient cases that had to be worked through before taking an exam or receiving credit for a class and video tutorials whose content was intended as preparation for seminars or considered to be relevant for an exam. In addition, seven teachers taught courses in which the use of Internet-based media was assigned great value in their teaching philosophies. For example, in one physical examination course an inverted classroom model was applied in the form of an online-based learning platform on which students could access literature, educational films and multiple-choice questions. This information was made available prior to the classes and used as the starting point for the classroom teaching allowing the course itself to serve as an opportunity for practical exercise [24].

#### Resources

**Figure 3:**
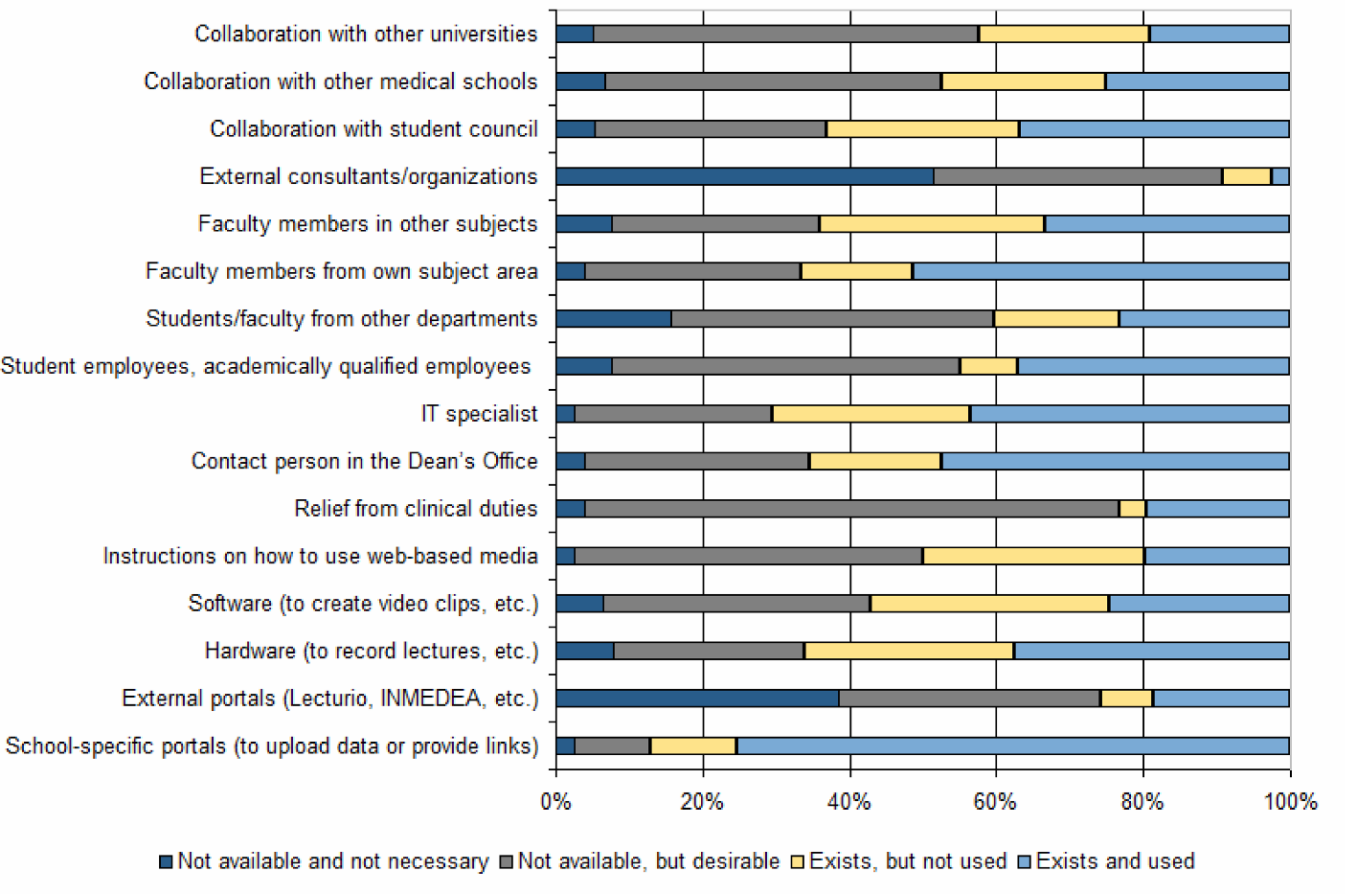
Organizational and staff resources available to teachers for using web-ased media and how these were put to use

**Figure 4:**
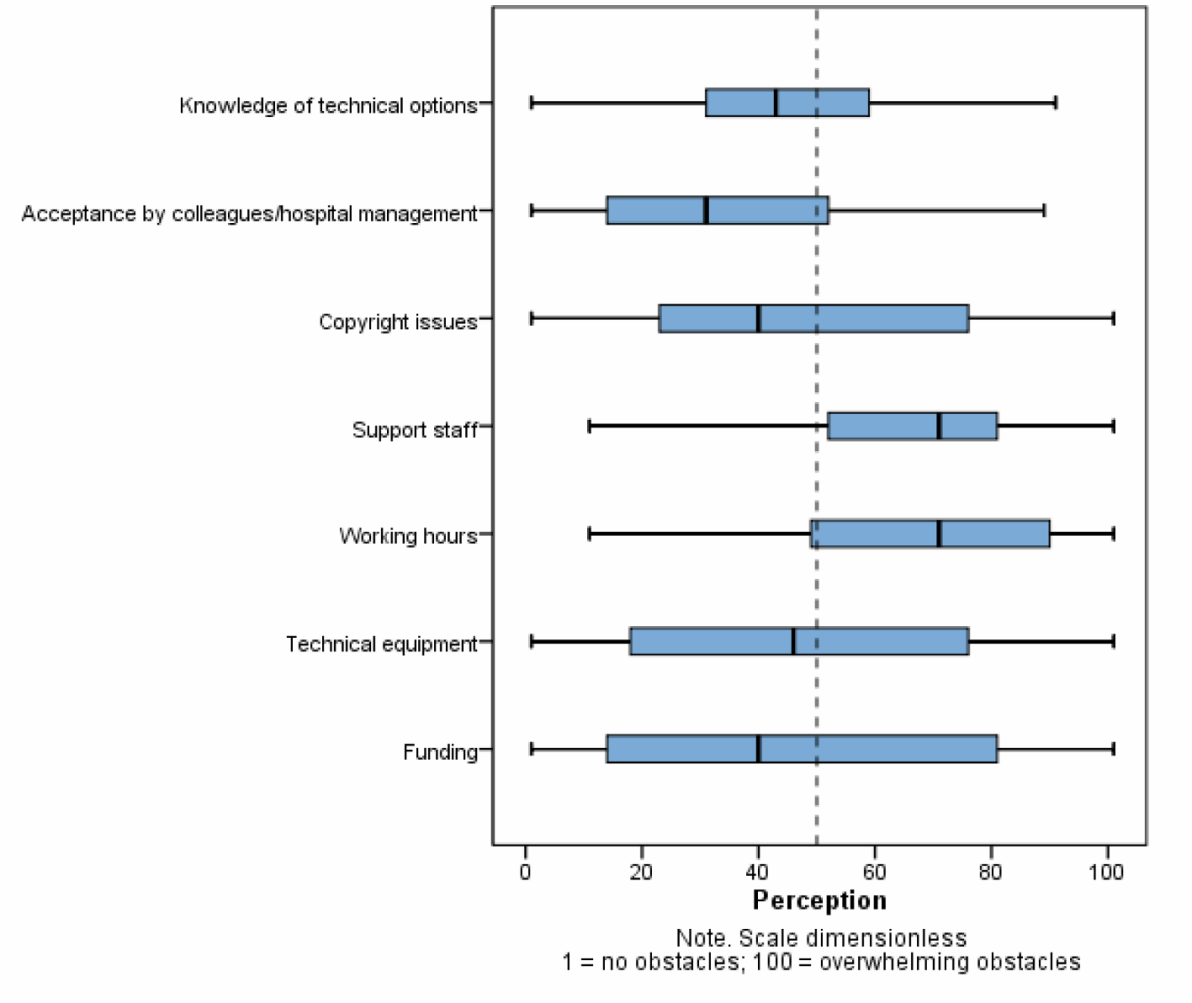
Perception of potential obstacles when web-based media were introduced into courses

Figure 3 (Fig. 3) shows which organizational and staff resources were available to the teachers for using web-based media and how these were then put to use. In addition to the available resources, 72% of the participants were able to take advantage of the expertise of a specialized department or interdisciplinary working group focused on e-learning and/or Internet-based media. In regard to funding, 38% of those surveyed indicated that they used hospital monies, 36% project funding from the medical school or university, and just as many reported using no special funding. Public funding, for instance grants, was used by 7% of the participants. How the significance of potential obstacles to implementation was perceived is presented in figure 4 (Fig. 4). A significant correlation was found between the extent of staff resources and the scope of web-based media use (*r_s_* (78)0.470; p<0.01) [25].

## 4. Discussion

This study is the first quantitative analysis of the range of Internet-based media in medical education in the German-speaking countries. It shows that web-based media still play a minor role in medical education in contrast to many other areas of life indicating that the actual importance of web-based media is overestimated in the published literature. Current publications on this topic are primarily overviews summarizing examples of application and current developments [14], [15], [16], [17]. An uncritical transference of these reviews, which predominantly cite Anglo-American studies and thus suggest a larger role for Internet-based media, to the German-speaking context makes little sense.

Our sample can be considered suitable for this study. Teacher coordinators were contacted in a targeted manner so that the teaching situation at each site could be extensively assessed. Even if a teacher expressed an unusual opinion, it cannot be assumed that the teacher differed significantly from the other teachers according to the personal characteristics covered by this survey, with the exception of teacher qualifications. The age distribution encompassed teachers who had access to web-based media as students in 2005 as well as those who were already older at that time thus covering a wide spectrum of experience. This is fully plausible in light of studies with larger samples [7], [26]. Participants represent both conventional and model curricula bringing aspects of both to bear. The response rate of 34.8% can be considered acceptable based on Cook et al. (2000) [27].

### Prevalence of Internet-based media

With only one to 28% of teachers actively using a particular Internet-based medium in classroom sessions, it is impossible to assume widespread use. Even if, when viewed from another angle, 50% of the participants are actively using at least one Internet-based medium, this mainly involves podcasts, interactive patient cases, and services to upload data. These tools are seen primarily as supplements to traditional classroom teaching. That only eight teachers (9.6%) integrate web-based media into the teaching and learning process in mandatory manner emphasizes the rarity of this even more strongly. Even if, as we suspect, web-based media are significantly more prevalent in the learning materials made available to students than they are in active classroom interactions, their presence in the form of those materials is also very limited. Although 94% of teachers upload lecture slides and 70% make use of podcasts, more active media such as interactive patient cases (34.5%), online-based learning programs (30.1%) and Wikipedia-like summaries (27.4%) follow far behind, despite the fact their use as teaching materials has many advantages. For instance, once these media have been developed they may be used repeatedly with little updating or monitoring and can be accessed via all common web-enabled devices regardless of time or place making them very effective for both students and teachers in terms of time [28]. In addition, virtually all of those surveyed have access to portals for uploading data. Despite rapid developments in Internet-based media, medical education remains far behind the current possibilities and the demands for modernization. New concepts for using modern media have not been developed to any measurable extent, but rather in most cases are only used to make existing classroom materials available in digital form. One reason for this can be found in the observation that the use of digital media in various studies has shown that there is only a moderate to slight effect on student performance and thus the relationship between amount of effort and beneficial result is open to critical questioning – recently this was again shown by Schneider & Preckel in their review of different meta-analyses [29].

If the use of social media such as Facebook, Twitter and blogs is taken into account, then these are clearly and significantly even less prevalent. However, several points must be considered. Negative aspects, particularly in regard to Facebook, are increasingly being identified in the literature, among them the potential lack of professionalism when using the medium, the wide-ranging consequences of careless postings, and student hesitation to participate in large-group discussions [6], [30], [31]. It must also be acknowledged that social media involve public spaces in which data privacy cannot be wholly ensured and users have no final control over how the social media provider exploits their personal data. Also, teachers and students are usually required to register for the platforms and consent to do this cannot be generally assumed [6]. So it is highly conceivable that only very few teachers use social media themselves and that most view such media as very poorly suited for use in teaching.

#### Influences on extent of use

The correlation seen between the information sources consulted on web-based media and the extent of web-based media use indicates that it is primarily teachers who are interested in this topic who include such media more extensively in their teaching. This also means that prior to using web-based media in teaching a substantial process of gathering information is required for which the teachers must have sufficient motivation. It is possible to assume that the assessment of an online-based medium’s suitability for teaching also plays a decisive role in its use in education. However, it is interesting that even media which are viewed as highly suitable, such as subject-specific apps and interactive patient cases are in reality only used to a very limited extent. There are many university teachers who do consider these media to have educational value, but do not make use of them. This discrepancy holds sufficient potential which could be harnessed through better organizational support, such as clarification of copyright issues and staff resources (see table 3 (Tab. 3)). Whether or not a teacher finds a web-based medium to be suitable for use in teaching is not dependent on the teacher’s own use of the medium, with the exception of Wikipedia. One reason for this is that few teachers use web-based media frequently or at all. No correlations were found between the basic attitude of the teachers toward web-based media and active use of them in classroom teaching. Teachers’ interest in actually putting web-based media to educational use in the classroom appears to be more important than how open or confident they are concerning web-based media or if they themselves use such media.

#### Available resources

From the teachers’ perspective, the availability of staff resources is in need of the most improvement. While only 42% of teachers have student assistants and 21% receive relief from clinical duties, other available resources such as hard- and software, training sessions and IT experts go in part unused or cannot be used. This primarily involves the great amount of time and effort required and a lack of support, and these represent the basic obstacles to implementation. Even the positive correlation between the extent of staff support and extent of Internet-based media use shows that the use of web-based media is a task that must be borne by multiple people if it is to be successful. With the growing reach of web-based media this will heavily depend in the future on the efficient deployment of resources and the creation of synergetic effects. One option that has been little utilized up to now (see figure 3 (Fig. 3)) is collaboration among teachers from different academic departments and universities. The creation of viable structures is critical, however, to transfer competence [32]. Contrary to expectations, for most participants a lack of funding did not pose a significant obstacle to using web-based media in education, so that optimistically it appears that schools are indeed providing sufficient funding to teachers for educational projects.

#### Limitations

The small sample size resulting from the low response rate along with the narrowed focus of the survey on clinical subjects limit the ability to generalize the study results. Moreover, a bias shown by pioneering medical schools cannot be ruled out since university affiliation was not recorded for reasons of data privacy. In addition, the discriminatory power of the items must be scrutinized to point out differences between users and nonusers and why actual use of Internet-based media in teaching could be even lower still. These limitations, however, do not contradict the basic observation of this study that Internet-based media continue to be given little importance in medical education.

## 5. Conclusions

Despite the rapid development of the Internet over the past 15 years, web-based media still play a subordinate role in the clinical phase of medical education. Primarily social media are considered unsuitable for teaching and even media which are viewed more favorably from an educational standpoint are only rarely used in classroom teaching or as learning tools. In view of the wide reaching influence exerted by such media on Generation Z, it will be necessary even in medical education to acknowledge these changes in daily life and to adapt and develop existing curricula in response [13], [33]. To accomplish this, a high level of interest on the part of teachers and the provision of sufficient staff resources are crucial. Future studies should investigate student expectations concerning the use of new media. New methods should also be identified to enable effective teacher networks extending across universities so that knowledge and experience with using web-based media can be shared.

## Competing interests

The authors declare that they have no competing interests. 

## Supplementary Material

Questionaire

## Figures and Tables

**Table 1:**
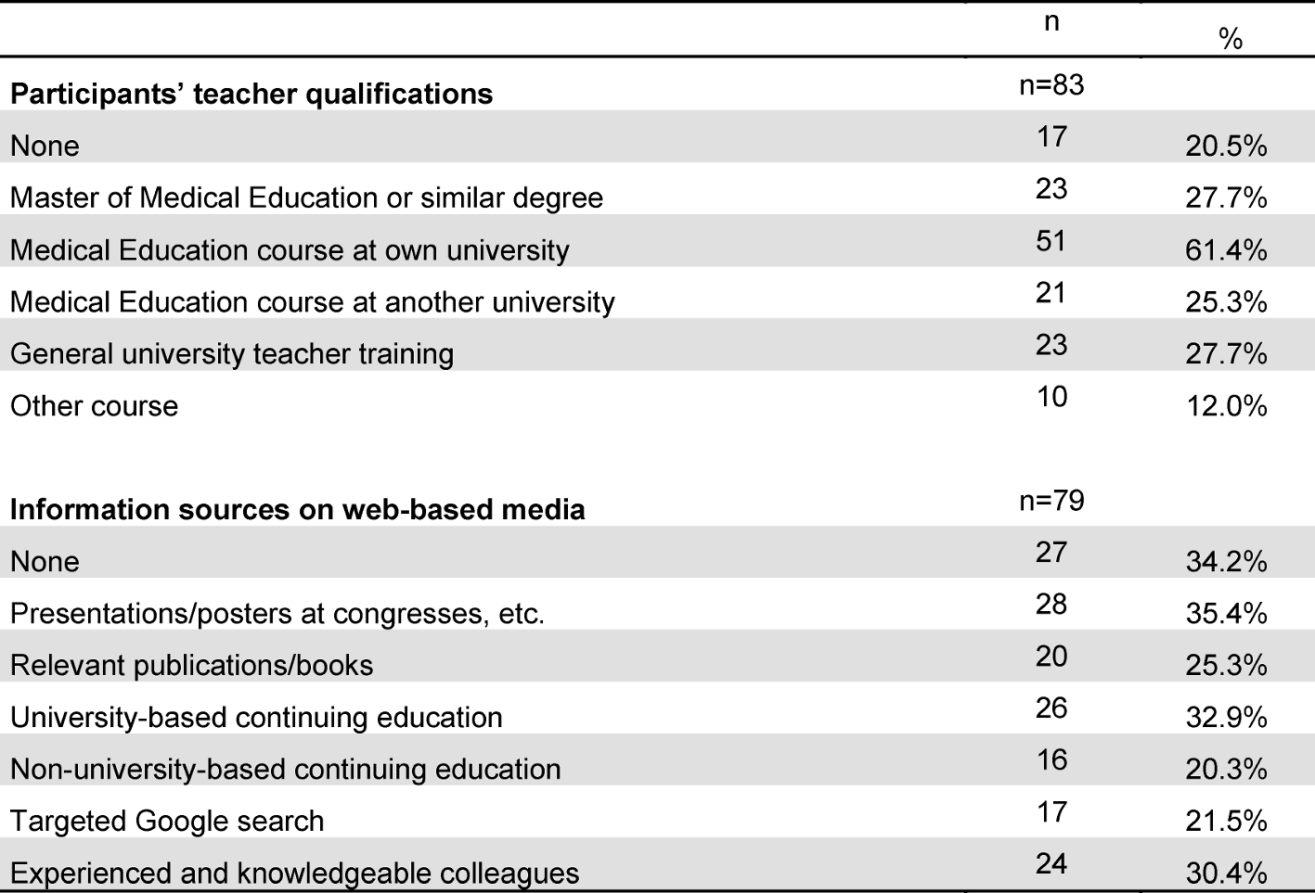
Professional teaching qualifications and information sources consulted

**Table 2:**
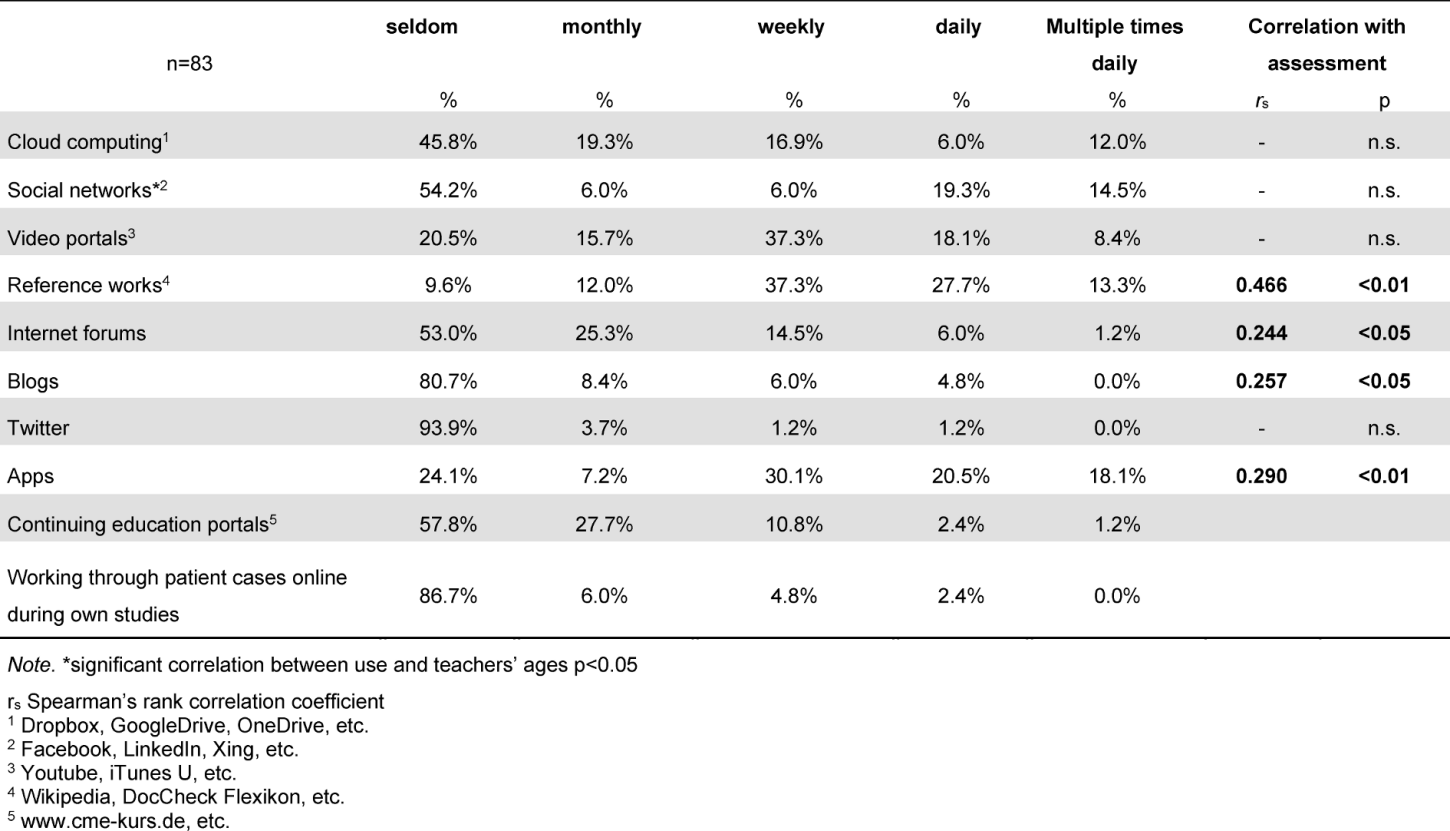
Frequency of personal use of web-based media and its correlation with assesment of educational value (see figure 1)

**Table 3:**
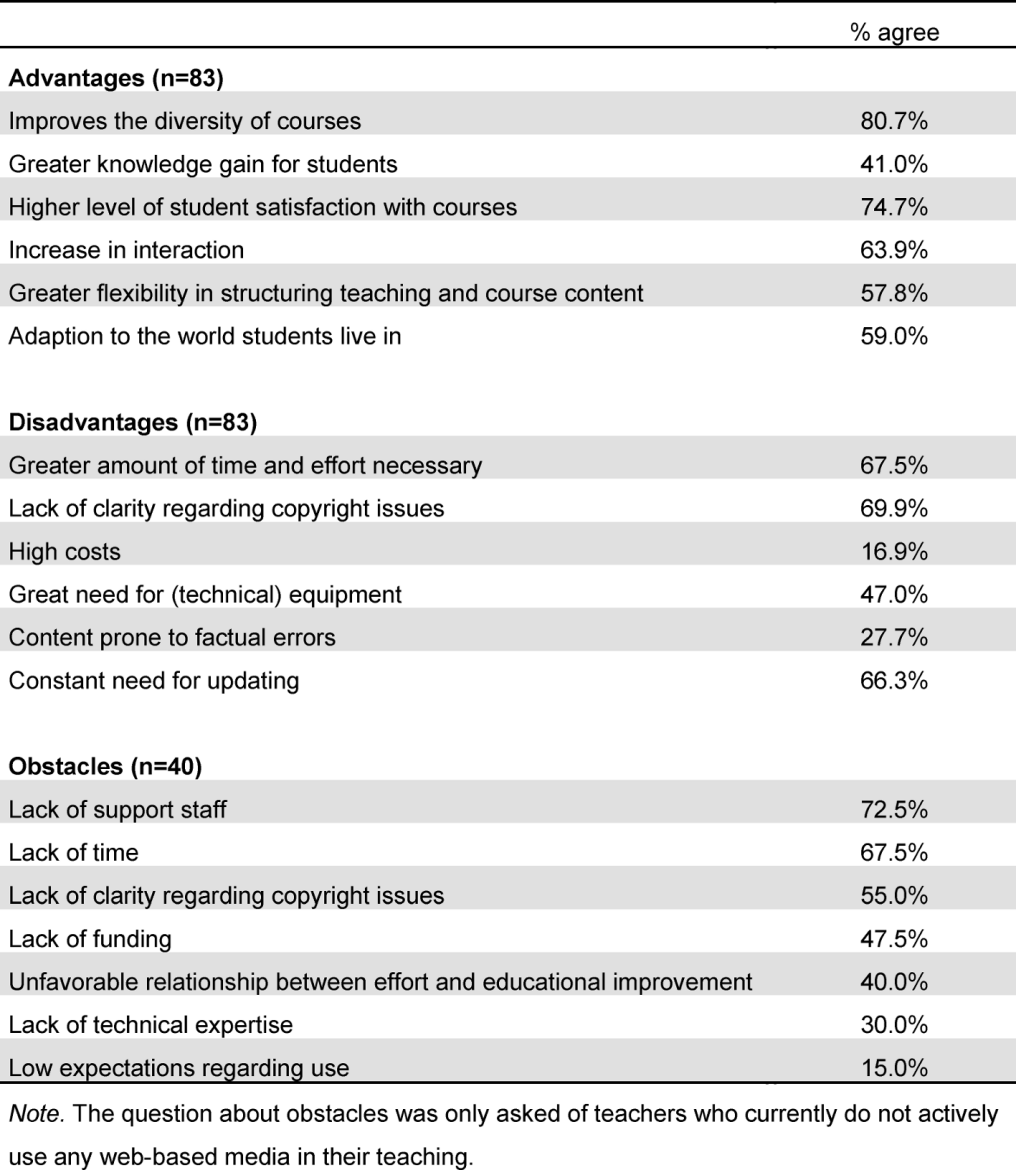
Advantages and disadvantages of using web-based media and the main obstacles to their use
