# Supplementary material for: Medical Education for “Generation Z”: Everything online?! – An analysis of Internet-based media use by teachers in medicine
Source: GMS J Med Educ. 2018 May 15;35(2):Doc21. doi: 10.3205/zma001168 (PMC6022581; doi:10.3205/zma001168)
Supplement: Questionaire [file JME-35-21-s-001.pdf]

Please fill out some general information about yourself. This information will remain anonymous.

1. Where is your medical school located?

O Germany   O Austria   O Switzerland

2. What is your subject specialty

O Anesthesia   O Surgery   O Internal Medicine   O Pediatrics
O Gynecology   O Psychiatry   O Other

3. What type of curriculum does your medical school have?

O Conventional curriculum   O Model curriculum   O Unknown

4. Which of the post-graduate programs in Medical Education listed below have you participated in? (Multiple answers are possible.)

O None
O Master of Medical Education or similar degree
O Medical Education course at own university
O Medical Education course at another university
O General university teacher training
O Other course:

5. What is your age?

O 20-29 years   O 30-39 years   O 40-49 years   O 50-59 years   O 60-69 years

The first section of the questionnaire addresses basic questions about teaching within your specialty subject.

6. Which teaching formats are used in your curriculum? (Multiple answers are possible.)

O Lectures
O Seminars
O Student-led tutorials
O Hospital/surgical clerkships (bed-side teaching)
O PBL courses
O Skills lab training
O Prescribed self-study
O Other:

7. Which formats are used for extra-curricular courses? (Multiple answers possible.)

O No extra-curricular courses exist
O Lectures
O Seminars
O Student-led tutorials
O Hospital/surgical clerkships (bed-side teaching)
O PBL courses
O Skills lab training
O Teacher-led self-study (interactive patient cases online, etc.)
O Other:

8. What type of teaching materials do you provide your students? How often are these used in your opinion?

	extremely often	Very often	Often	Moderately	Seldom	Very rarely	Never	Not applicable
Lecture slides as downloadable PDFs	O	O	O	O	O	O	O	O
Lecture notes	O	O	O	O	O	O	O	O
Old tests as downloadable PDFs	O	O	O	O	O	O	O	O
Video clips of lectures	O	O	O	O	O	O	O	O
Audio recordings of lectures	O	O	O	O	O	O	O	O
Educational films made by you (practical skills, etc.)	O	O	O	O	O	O	O	O
Educational films from another source (YouTube, etc.)	O	O	O	O	O	O	O	O
Interactive patient cases online	O	O	O	O	O	O	O	O
Links to the university library's ebook collection and other resources	O	O	O	O	O	O	O	O
Relevant publications as links or downloads	O	O	O	O	O	O	O	O
Relevant guidelines as links or downloads	O	O	O	O	O	O	O	O
Brief summaries (similar to Wikipedia) on important course content	O	O	O	O	O	O	O	O
Online-based learning programs	O	O	O	O	O	O	O	O
Other:	O	O	O	O	O	O	O	O

9. Which channels of communication are available to students when they wish to contact a teacher in your subject? How often are these used in your opinion?

	extremely often	Very often	Often	Moderately	Seldom	Very rarely	Never	Not applicable
Visit to the secretarial office	O	O	O	O	O	O	O	O
Teachers' office hours / personal contact	O	O	O	O	O	O	O	O
Telephone	O	O	O	O	O	O	O	O
Email	O	O	O	O	O	O	O	O
Internet forum	O	O	O	O	O	O	O	O
Blogs	O	O	O	O	O	O	O	O
Comment functions	O	O	O	O	O	O	O	O
Social networks	O	O	O	O	O	O	O	O
Other:	O	O	O	O	O	O	O	O

The use of web-based media in medical education depends on teachers and teacher coordinators. For this reason we are interested in your personal experiences, attitudes and opinions regarding web-based media.

10. How often do you use the following web-based media on a personal basis?

	Multiple times daily	Daily	Weekly	Monthly	Less often
Platforms like DropBox or GoogleDrive (to upload and share data)	O	O	O	O	O
Social networks (Facebook, Xing, Google+, etc.)	O	O	O	O	O
Video portals (YouTube, MyVideo, iTunes U, etc.)	O	O	O	O	O
Reference works (Wikipedia, DocCheck Flexicon)	O	O	O	O	O
Internet forums	O	O	O	O	O
Blogs	O	O	O	O	O
Microblogs, Twitter	O	O	O	O	O
Apps for mobile devices	O	O	O	O	O
Online portals like cms-kurs.de to accrue CE credits	O	O	O	O	O
Interactive patient cases online during own medical study	O	O	O	O	O

11. To what extent do you agree with the following statements?

	Agree completely	Mostly agree	Undecided	Mostly disagree	Disagree completely
I inform myself as often as possible about technical innovations, such as smartphones, computers, software, Internet applications.	O	O	O	O	O
I often consider how I can use technical innovations.	O	O	O	O	O
If I am interested in a technical innovation, then I inform myself in detail about it.	O	O	O	O	O
Friends and acquaintances describe me as being interested in technology.	O	O	O	O	O

Working with social media makes me nervous.	O	O	O	O	O
The thought that data could be lost if a mistake is made while using social media frightens me.	O	O	O	O	O
I am afraid I will make mistakes I can't fix when using social media.	O	O	O	O	O
I am somewhat intimidated by social media.	O	O	O	O	O
I am concerned that data I post on the Internet could be misused.	O	O	O	O	O
When I make purchases online, I am concerned that my credit card information could be stolen.	O	O	O	O	O
I don't like to post information online because I don't know how others might use it.	O	O	O	O	O
I don't like to publish information online because it could be stolen in ways I can't foresee.	O	O	O	O	O
I am familiar with the terms of use for the Web 2.0 tools that I use.	O	O	O	O	O
I worry that unauthorized persons could access my online data.	O	O	O	O	O

**12. Please respond with 1 (agree completely) to 5 (disagree completely).
I am able to complete a task using web-based media,**

	Agree completely	Mostly agree	Undecided	Mostly disagree	Disagree completely
Even if no one is there to tell me how to do it.	O	O	O	O	O
If I can ask someone for help when I get stuck.	O	O	O	O	O
If I had enough time.	O	O	O	O	O
Even if I am only able to use the integrated help function on the platforms for assistance.	O	O	O	O	O

This next section mainly covers the active use of web-based media in your teaching practices. Active use refers to direct use of web-based media in classroom teaching.

13. Which advantages do you see in using web-based media in classroom teaching? (Multiple answers are possible.)

O Improves the diversity of courses
O Greater knowledge gain for students
O Higher level of student satisfaction with the courses
O Increased interaction
O Flexibility
O Adaption to the world students live in
O Other advantages:

14. Which disadvantages do you see in using web-based media the classroom? (Multiple answers are possible.)

O More time and effort required
O Lack of clarity regarding copyright issues
O Expense
O Great need for (technical) equipment
O Content prone to factual errors
O Constant need for updating
O Other disadvantages:

15. How well suited are the following web-based media for teaching in your subject area? Which of these media are used directly in your teaching?

	1 (unsuited) to 100 (excellently suited)	Used by us
Podcasts (audio/visual recording of lectures)		
Social networks (Facebook, Xing, etc.)		
Microblogs (Twitter, etc.)		
Video conferences		
Cloud computing (common platforms to share data)		
Blogs		
Forums		
Interactive patient cases		
Wikis (Wikipedia, DocCheck Flexicon)		
Subject-specific applications for mobile devices		

Question 16.1 and 17.1 were asked only of respondents who did not mark at least one medium as "used".

16.1 Which reasons not to use web-based media in the classroom have been persuasive for you? (Multiple answers are possible.)
O Lack of time
O Low expectations of benefits
O Lack of technical expertise
O Lack of funding
O Lack of support staff
O Lack of clarity regarding copyright issues
O No improvement in teaching or learning is to be expected
O Students show good self-initiative in finding and using appropriate media
O The effort required is too high for the expected educational improvement (efficiency)
O Other reasons:

17.1 What would motivate you to use web-based media in a targeted manner in the classroom? (Multiple answers are possible.)
O Provision of funding
O Published papers pointing out the benefits of using web-based media
O Clear desire on the part of students to work with web-based media
O Innovative ideas from fellow teachers
O Support in the form of trained consultants, perhaps by a specialized department within the medical school
O Provision of staff for implementation
O Other motivations:

16. Do you hold seminars in which the use of web-based media is required of students (e.g. interactive patient cases)?	
O Yes	O No
**17. Please explain briefly in which way web-based media is integrated into your course.**	
	No mandatory use

18. Do you teach seminars or other kinds of classes that specifically focus on using web-based media?	
O Yes	O No
**19. Please describe the course design.**	
	No applicable courses

The final section asks which prerequisites are necessary for successful integration of web-based media into the educational process and which obstacles can interfere with their use.

20. Which organizational resources for using web-based media are available to you from your department or medical school? Which resources do you wish were available and which do you consider unnecessary?				
	Exists and used	Exists, but not used	Not available, but desirable	Not available and not necessary
School-specific portals (to upload data or provide links)	O	O	O	O
External portals (Lecturio, INMEDEA, etc.)	O	O	O	O
Hardware (to record lectures, etc.)	O	O	O	O
Software (to create video clips, etc.)	O	O	O	O
Instruction on how to use web-based media	O	O	O	O
Relief from clinical duties	O	O	O	O
Personnel (student/research assistants, IT staff)	O	O	O	O

21.Which types of funding do you use to provide specific web-based media in your courses? (Multiple answers are possible.)	
O No special funding	O Public funding (state level)
O Project funding from medical school/university	O Grants, funding from charitable trusts
O Hospital monies	O Other:
O Funding from private sector	

22. What kinds support staff are available to you to implement specific types of courses (e.g. creating new teaching materials such as videos, etc.)? What types of support would you like to have and what types do you find unnecessary? (Multiple answers are possible.)				
	Exists and used	Exists, but not used	Not available, but desirable	Not available and not necessary
Contact person in the Dean's Office	O	O	O	O
IT specialist	O	O	O	O
Student employees, academically qualified employees at your hospital	O	O	O	O
Student employees, academically qualified employees from other departments (e.g. Dean's Office)	O	O	O	O
Faculty members from own subject area	O	O	O	O
Faculty members in other subjects	O	O	O	O
External consultants/organizations	O	O	O	O
Collaboration with student council	O	O	O	O
Collaboration with other medical schools	O	O	O	O
Collaboration with other universities	O	O	O	O
Other:	O	O	O	O

**23. Does your medical school have a department or interdisciplinary working group dedicated to e-learning and Internet-based media?**		
O Yes	O No	O Unknown

**24. How much effort do you put forth to integrate web-based media in your courses?**
1 (extremely little) to 100 (extremely high)

**25. Where do you encounter large obstacles or none at all when introducing the use of web-based media into your courses?**	
	1 (no obstacles) to 100 (overwhelming obstacles)
Funding	
Technical equipment	
Working hours	
Support staff	
Copyright issues	
Acceptance by colleagues/hospital management	
Knowledge of technical options	
Other:	

**26. Which sources of information or opportunities for continuing education have you already taken advantage of? (Multiple answers are possible.)**	
O None	O Non-university-based continuing education
O Presentations/posters at congresses or other events	O Targeted Google search
O Relevant publications/books	O Experienced and knowledgeable colleagues
O University-based continuing education	OOther:
